# Paraquat-Induced Reactive Oxygen Species Inhibit Neutrophil Apoptosis via a p38 MAPK/NF-κB–IL-6/TNF-α Positive-Feedback Circuit

**DOI:** 10.1371/journal.pone.0093837

**Published:** 2014-04-08

**Authors:** Xiaolong Wang, Fuling Luo, Hengguang Zhao

**Affiliations:** 1 Emergency department, the 2nd affiliated hospital of Chongqing Medical University, Chongqing, China; 2 Department of Pharmacy, the First Affiliated Hospital of Chongqing Medical University, Chongqing, China; 3 Department of Dermatology, the First Affiliated Hospital of Chongqing Medical University, Chongqing, China; University of California, Merced, United States of America

## Abstract

Paraquat (PQ), a widely used herbicide and potent reactive oxygen species (ROS) inducer, can injure multiple tissues and organs, especially the lung. However, the underlying mechanism is still poorly understood. According to previous reports, neutrophil aggregation and excessive ROS production might play pivotal pathogenetic roles. In the present study, we found that PQ could prolong neutrophil lifespan and induce ROS generation in a concentration-independent manner. Activated nuclear factor-κB (NF-κB), p38 mitogen-activated kinase (p38 MAPK), and myeloid cell leukemia sequence 1 (Mcl-1) but not Akt signaling pathways were involved in this process, as well as increasing levels of interleukin-6 (IL-6), tumor necrosis factor-α (TNF-α), and IL-1β. Furthermore, the proinflammatory mediators IL-6 and TNF-α could in turn promote ROS generation, creating a vicious cycle. The existence of such a feedback loop is supported by our finding that neutrophil apoptosis is attenuated by PQ in a concentration-independent manner and could partially explain the clinical dilemma why oxygen therapy will exacerbate PQ induced tissue injury.

## Introduction

The nonselective contact herbicide paraquat (PQ) is a strong pneumotoxicant; it accumulates in the lung through a polyamine uptake system and can induce redox cycling, leading to oxidative stress-related damage [1], [2]. Evidence has shown that reactive oxygen species (ROS) occupy a central role in PQ-induced acute lung injury (ALI). ROS production impairs tissue and cell function by inducing lipid peroxidation, protein damage, and DNA breakage [3]. In addition to direct ROS-induced pathology, the inflammation response that is secondary to PQ poisoning is also involved in disease pathogenesis. As intrinsic signal transduction molecules, ROS are important components in the complex modulation of neutrophil apoptosis [4], [5]. Under normal circumstances, rapid apoptosis is a neutrophilic characteristic. The average life span of neutrophil is 8-20 h in circulation [6]. Control of neutrophil apoptosis is essential to rapidly resolve inflammatory reactions [7]. Recent studies have shown that apoptosis contributes to ALI pathogenesis, and neutrophil apoptosis in particular exacerbates the condition [8]–[11].

Studies have shown that ROS are involved in promoting cell survival or apoptosis in concentration- and cell type-dependent fashions. Different ROS levels may have opposite effects in the same type of cell [12]. Accordingly, the role of ROS in neutrophil apoptosis remains controversial. One study demonstrated that antioxidants indirectly delay the onset of apoptosis, which suggested that oxidative stress promotes neutrophil apoptosis [13]. On the other hand, several groups have shown that oxidants could delay neutrophil apoptosis, which in turn generated more ROS [14]–[16].

Several studies have demonstrated that ROS are involved in modulating the nuclear translocation and transcriptional activity of nuclear factor κB (NF-κB), and inhibitors of ROS can block NF-κB activity [17]–[19]. In addition, ROS can promote NF-κB nuclear accumulation in certain cell lines [18]. NF-κB activation plays an important role in delaying neutrophil apoptosis, resulting in the release of prosurvial inflammatory factors, including macrophage inflammatory protein-2 (MIP-2) and tumor necrosis factor-α (TNF-α) [20]. However, there are some paradoxical reports that ROS does not mediate NF-κB activation [21]–[23]. Some experiments have shown that ROS participate in the phosphorylation of p38 mitogen-activated kinase (p38 MAPK) [24], [25], which can enhance myeloid cell leukemia sequence 1 (Mcl-1) stabilization. We further investigated the effects of PQ on neutrophil apoptosis and found that it prolonged neutrophil lifespan in a concentration-independent manner, and this delay of neutrophil apoptosis might, at least partly, through its effects on a positive feedback circuit involving p38 MAPK, NF-κB, IL-6, and TNF-α.

## Materials and Methods

### Human neutrophil isolation and culture

The project was approved in accordance with the ethical committee approval process of the First Affiliated Hospital of Chongqing Medical University. After a written informed consents were obtained, peripheral blood neutrophils from healthy human donors were isolated by dextran sedimentation followed by double density gradient centrifugation in Histopaque-1119 and Histopaque-1117 separation media (Sigma, St. Louis, MO, USA) as described previously [19]. Contaminating erythrocytes were lysed by hypotonic shock with 0.2% NaCl, and the final viability of isolated neutrophils was assessed by trypan blue dye (routinely >98%). Neutrophils were then resuspended at a density of 1.6×10^5^/ml in Dulbecco's phosphate-buffered saline (DPBS) with Ca^2+^, Mg^2+^, 1 g/L glucose, and 4 mM sodium bicarbonate.

### Treatment

To detect the effect of PQ on neutrophil survival, neutrophils were stimulated with PQ (1, 5, 50, or 100 μM) for 6 to 24 h. Diphenyleneiodonium (DPI, 50 μM, Sigma) and apocynin (10 μM, Sigma), the specific inhibitors of NADPH oxidase (Nox), as well as pyrrolidine dithiocarbamate (PDTC, 10 μM, Sigma), SB203580 (20 μM, Sigma), SC 200137 (25 μM, Sigma), and SC 221226 (20 μM, Sigma), the specific inhibitors of NF-κB, p38 MAPK, Mcl-1, and Akt, respectively, were added 30 min before PQ treatment. Normal saline was used as the negative control.

### Detection of ROS

Neutrophil cytosolic ROS levels were investigated with an OxiSelect Intracellular ROS Assay Kit (Cell Biolabs, San Diego, CA, USA) according to the manufacturer's instruction. Briefly, after the culture media was removed, cells were gently washed with DPBS 3 times. Then, 100 μL 1× dichloro-dihydro-fluorescein diacetate (DCFH-DA) solution was added into the cells and incubated at 37°C for 45 minutes. Cells were again washed with DPBS 3 times. The fluorescence intensities of dying cells were then assessed by flow cytometry at 488-nm excitation wavelength.

### Analysis of neutrophil apoptosis

Neutrophil apoptosis was measured with an annexin V-fluorescein isothiocyanate (FITC)/propidium iodide (PI) apoptosis assay kit (Pharmingen, BD Biosciences, San Diego, CA, USA) followed by flow cytometry. Briefly, cultured neutrophils were washed twice with ice-cold PBS and resuspended in binding buffer. Annexin V-FITC and PI were then added following the manufacturer's instructions, and apoptotic neutrophils were analyzed by flow cytometry within 1 h after labeling. Apoptosis was defined as positive annexin V-FITC labeling but negative PI staining. Results are expressed as a percentage of apoptotic neutrophils relative to the total amount of counted neutrophils.

### Cytokine measurement

At 6 h after 5 μM PQ stimulation, levels of TNF-α, IL-1β, and IL-6 in neutrophil supernatants were assayed with an ELISA kit (Biosynthesis Biotechnology, Beijing, China) following the manufacturer's instructions. For the inhibition experiments, polyclonal antibodies against TNF-α, IL-1β, and IL-6 were individually preincubated with neutrophils at final concentrations of 5-fold to the respective cytokines 30 min before PQ treatment.

### Western blotting

Proteins from cultured neutrophils were prepared with a protein extraction solution (containing 20 mmol/L Tris (pH 7.4), 150 mmol/L NaCl, 1 mmol/L EDTA, 1 mmol/L EGTA, 1% Triton, 0.1% SDS and 1% protease inhibitor cocktail). Protein concentrations were determined with a bicinchoninic acid (BCA) protein assay kit (Thermo Fisher, Waltham, MA, USA). Then, 40 μg samples of protein extracts were fractionated on 12% polyacrylamide-sodium dodecyl sulfate (SDS) gels and then transferred to nitrocellulose membranes. The membranes were blocked with 5% (w/v) fat-free milk in Tris-buffered saline (TBS) containing 0.05% Tween-20, followed by incubation with primary antibodies to phosphorylated-p38 MAPK (p-p38 MAPK), phosphorylated Mcl-1 (p-Mcl-1), Akt, phosphorylated P65 (p-P65), phosphorylated IκBα (p-IκBα), and α-tubulin (all at 1∶1000; Abcam, Cambridge, UK) at 4°C overnight. The next day, the membranes were incubated with horseradish peroxidase-conjugated secondary antibody (1∶5000, Abcam), and bands were visualized with an enhanced chemiluminescence system with short exposure to X-ray films (Kodak, Rochester, NY, USA).

### Statistical Analysis

All data are expressed as mean ± standard deviation (M ± SD) from four independent experiments. Results were analyzed by Student's t test or analysis of variance (ANOVA). *P* values≤0.05 were considered statistically significant.

## Results

### PQ delayed neutrophil apoptosis in a concentration-independent manner

**Figure 1:**
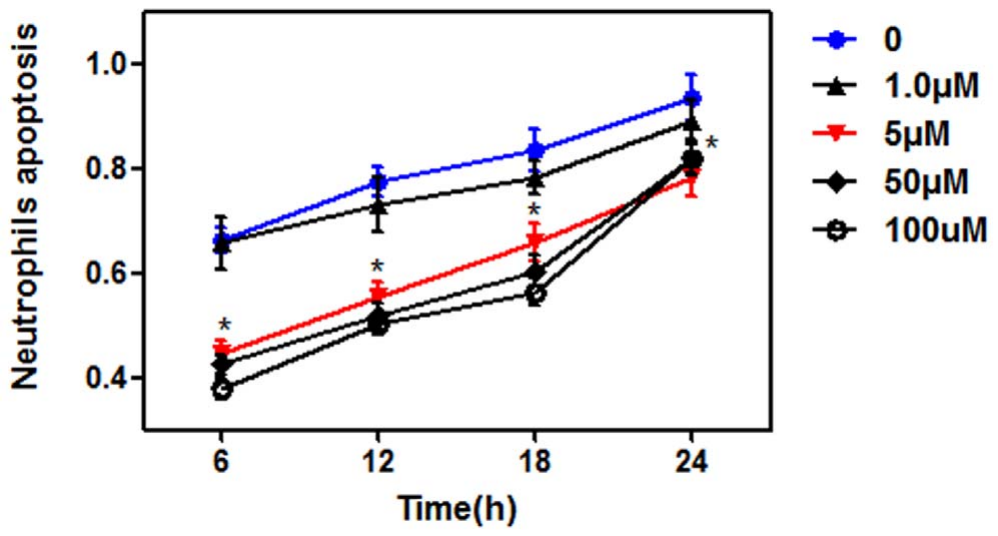
PQ delayed neutrophil apoptosis in a concentration-independent manner. Neutrophil apoptosis was assessed with annexin V-FITC and PI staining followed by flow cytometry. Compared with the control, the doses of PQ (5, 50, or 100 μM) significantly attenuated neutrophil apoptosis at all time points after treatment (6, 12, 18, and 24 h). No difference was found between different concentration groups. *P<0.05 compared with control at the same time point.

To investigate the effect of PQ on neutrophil apoptosis, Annexin V-FITC and PI labeling were assessed with flow cytometry. We found that stimulation with 1 μM PQ did not affect neutrophil survival, but 5, 50, or 100 μM PQ significantly delayed neutrophil apoptosis at all assessed time points (6, 12, 18 and 24 h after treatment) (Fig. 1). This showed that certain concentrations of PQ (≥5 μM) could prolong neutrophil survival in a concentration-independent manner. The highest percentage of delay was 6 h after treatment in each group. Hence, we used 5 μM PQ for 6 h in the next experiments.

### PQ-induced ROS generation regulates neutrophil apoptosis

**Figure 2:**
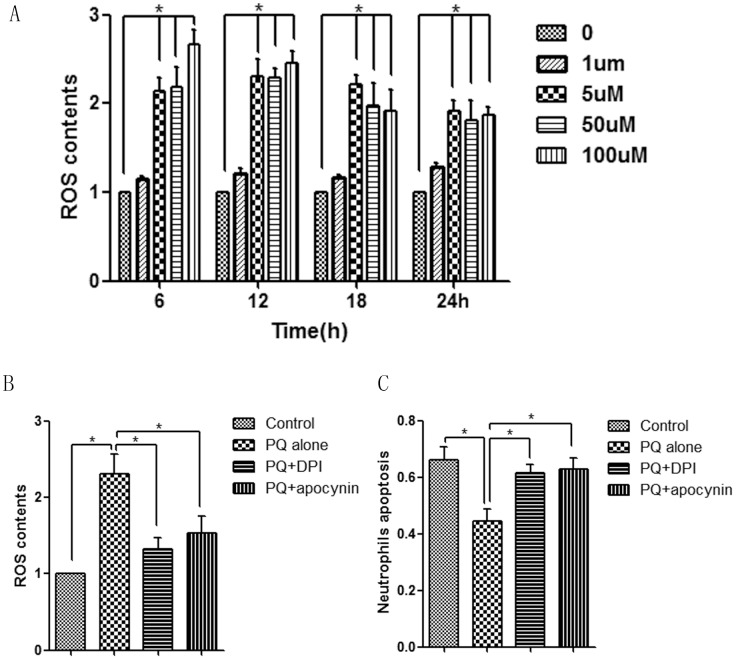
ROS were involved in PQ-induced neutrophil apoptosis delay. (A) ROS generation was assessed with DCFH-DA fluorescence intensity and was remarkably increased by all PQ concentration (5, 50, and 100 μM) at all time points (6, 12, 18, and 24 h) in a concentration-independent manner. (B) DPI or apocynin pretreatment suppressed PQ-induced ROS generation. (C) PQ-induced neutrophil apoptosis was also attenuated by DPI or apocynin pretreatment. ROS contents were normalized to control. *P<0.05.

As shown in Fig. 2A, PQ-induced ROS was investigated by DCFH-DA fluorescence intensity and flow cytometry at 6, 12, 18, and 24 h after treatment. Consistent with the observed effect of PQ on neutrophil apoptosis, 1 μM PQ showed no impact on ROS generation compared with the control group. However, ROS concentrations were markedly increased by 5, 50, or 100 μM PQ at all four time points (normalized to control group in Fig. 2A).

Physiologically, receptors in neutrophils stimulated by NADPH oxidases lead to oxidative bursts and ROS generation [26]. To further investigate whether PQ-induced ROS were the prosurvival factor in neutrophils, we applied DPI or apocynin, the NADPH oxidase-specific inhibitors, to block ROS productions. After 50 μM DPI or 10 μM apocynin pretreatment, ROS generation was decreased from 2.31- to 1.33- or 1.54-fold (normalized to control groupin Fig. 2B), and neutrophil apoptosis was elevated from 44.6% to 61.8% or 62.9% (Fig. 2C) compared with PQ 5 μM treatment alone. These results demonstrated that ROS were involved in the regulation of neutrophil apoptosis and that PQ delayed neutrophil apoptosis through activating the ROS pathway.

### p38 MAPK and NF-κB signaling pathways, but not Akt, were involved in PQ-induced ROS accumulation and delayed neutrophil apoptosis

**Figure 3:**
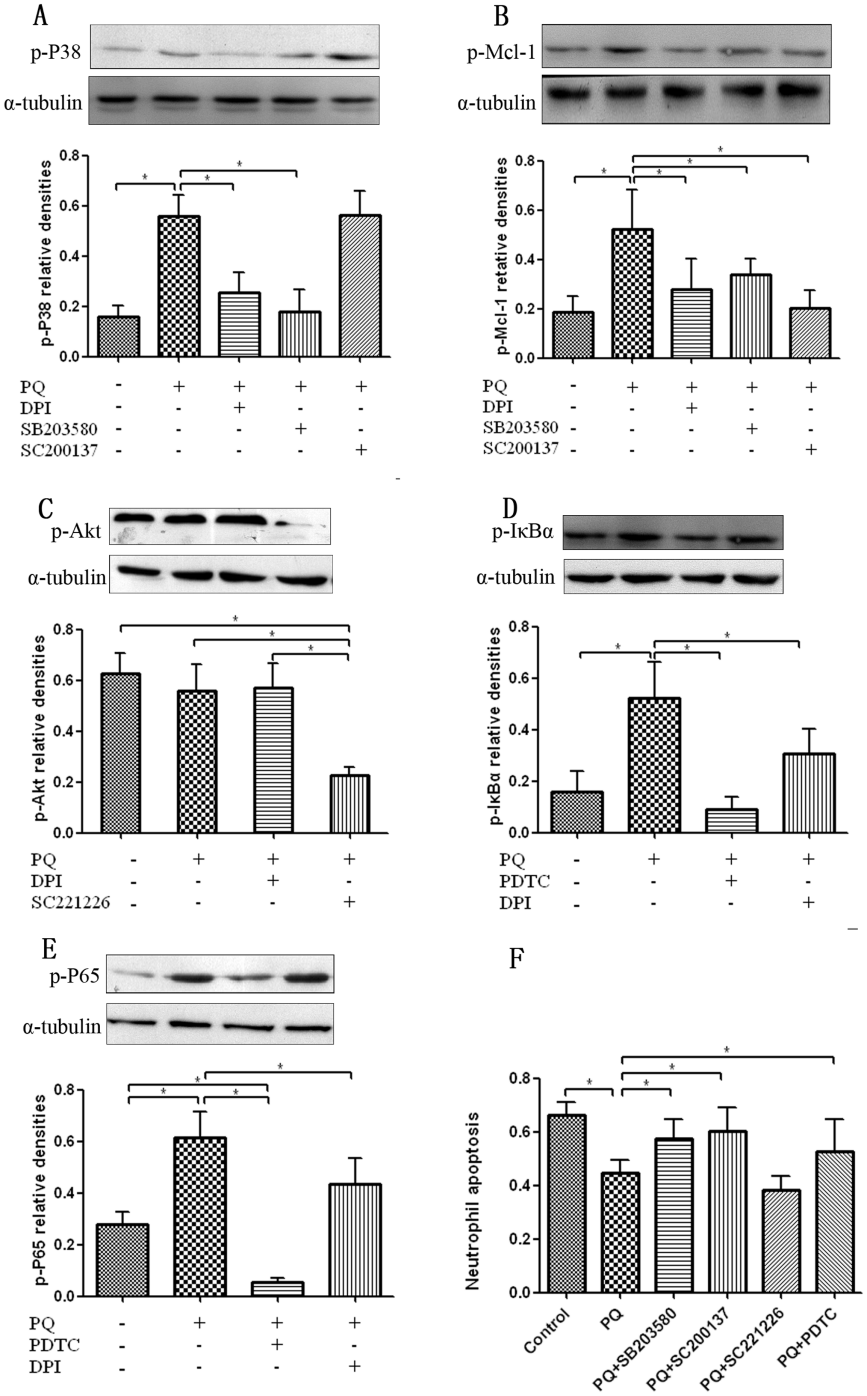
p38 MAPK and NF-κB signaling pathways, but not Akt, were involved in PQ-induced ROS generation and reduced neutrophil apoptosis. (A, B) p-p38 MAPK and p-Mcl-1 production were predominantly induced by PQ and suppressed by DPI co-treatment. The specific p38 MAPK inhibitor SB203580 blocked both p-p38MAPK and p-Mcl-1 productions, whereas SC200137 suppressed p-Mcl-1 but not p-p38MAPK. (C) The Akt pathway was not affected by PQ. (D, E) Both p-IκBα and p-P65 were activated by PQ pretreatment and decreased by DPI. (F) PQ-mediated reduction in neutrophil apoptosis could be rescued by SB203580, SC200137, and PDTC but not SC221226, which indicated that p38MAPK, Mcl-1, and NF-κB pathways were involved in the PQ-regulated decrease in neutrophil apoptosis. *P<0.05.

It is well established that p38 MAPK, Akt, and NF-κB signaling pathways promote the survival of granulocytes and lipopolysaccharide (LPS)-stimulated neutrophils[27], which subsequently affect cell lifespan[25], [28]. Therefore, we further analyzed these pathways. As shown in Fig. 3A and 3B, the expressions of p-p38 MAPK and p-Mcl-1 were predominantly induced by PQ at 6 h and were suppressed by DPI co-treatment. These results indicated that p38 MAPK and Mcl-1 were involved in PQ-induced neutrophil survival and that ROS are located upstream of p38 MAPK and Mcl-1 signaling pathways. To further elucidate the effects of p38 MAPK and Mcl-1, we applied SB203580 and SC200137, the specific inhibitors of p38 MAPK and Mcl-1, to block their respective activities. The results indicated that both SB203580 and SC200137 could reverse the PQ-induced increases in p-p38 MAPK and p-Mcl-1 production (Fig. 3A, 3B), as well as the effect on neutrophil survival (Fig.3F). SB203580 could block the production of both p-p38 MAPK and p-Mcl-1, but SC200137 could only suppress p-Mcl-1 (Fig. 3A, 3B), which further demonstrated that p38 MAPK and Mcl-1 were involved in a single signaling pathway, and Mcl-1 was located downstream of p38 MAPK.

As shown in Fig. 3C, PQ treatment did not affect Akt expression at 6 h, and pretreatment with its specific inhibitor, SC221226, also did not exert any obvious effect on neutrophil apoptosis (Fig. 3F).

Next, PDTC treatment was employed to block NF-κB signaling. The results showed that 5 μM PQ could promote the production of both p-IκBα (Fig. 3D) and its downstream effector p-P65 (Fig. 3E) at 6 h, while PDTC suppressed the antiapoptotic effect of PQ on neutrophils (Fig. 3F) through decreasing the expression of p-IκBα (Fig. 3D) and p-P65 (Fig. 3E). These findings indicated that the NF-κB pathway was also involved in the antiapoptotic effect of PQ on neutrophils. Meanwhile, elevations in p-IκBα (Fig. 3D) and p-P65 (Fig.3E) induced by PQ were both decreased following co-treatment with DPI, indicating that the NF-κB pathway was also involved in the regulation of PQ-induced ROS generation. Taken together, our results suggest that the NF-κB pathway is involved in the regulation of PQ-induced neutrophil apoptosis delay and that it is located downstream of the ROS pathway.

### TNF-α and IL-6, but not IL-1β, formed a positive feedback circuit with ROS generation upon PQ stimulus

**Figure 4:**
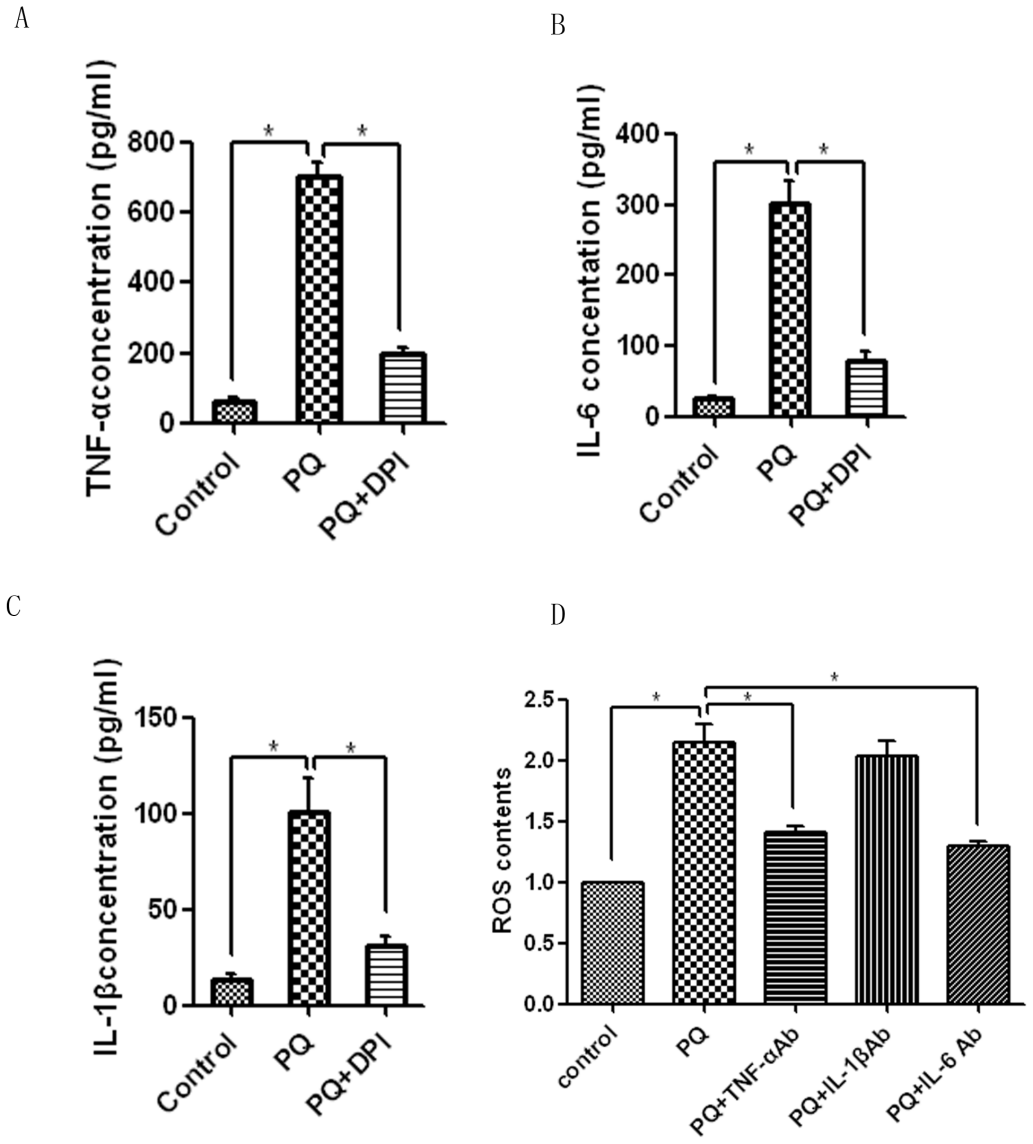
TNF-α and IL-6, but not IL-1β, formed a positive-feedback circuit with ROS generation upon PQ stimulus. (A) TNF-α, IL-1β, and IL-6 production levels were remarkably promoted by PQ and significantly decreased by DPI. (B) PQ-induced ROS generation was decreased by preincubation with TNF-α or IL-6 antibody respectively, but not by IL-1β, which indicated a positive-feedback circuit between the regulation of ROS generation and TNF-α and IL-6 production under PQ stimulus. ROS contents were normalized to control. *P<0.05.

Cytokines such as TNF-α, IL-1β, and IL-6 are involved in the early inflammatory phase of PQ poisoning [29]. However, the inherent relationship between these cytokines and ROS is still poorly understood. For this reason, we assessed TNF-α, IL-1β, and IL-6 levels in cultured neutrophil supernatant after PQ treatment. The results showed that PQ strongly promoted TNF-α, IL-6, and IL-1β expression, and compared with the PQ-alone group, DPI pretreatment significantly decreased levels of all three cytokines (Fig. 4A, B, C), which indicated that PQ induced the expression of these cytokines through the ROS pathway. Further, polyclonal antibodies against TNF-α, IL-6, and IL-1β were used as the antagonists. As shown in Fig. 4D, the levels of ROS induced by PQ were decreased by preincubation with TNF-α or IL-6 antibody, but not by an antibody against IL-1β. The results indicate that ROS was a potent stimulator of TNF-α and IL-6, which could then reciprocally promote ROS generation (ROS contents were normalized to the control group in Fig. 4D). A positive feedback circuit controlled ROS generation following PQ treatment, and this pathway might play a central role in the persistent injuries in PQ-poisoned tissues.

## Discussion

It is well known that PQ is a potent inducer of intracellular ROS, which have an essential role in PQ-induced tissue injury [30]–[31]. In addition to its direct effects on tissues [3], ROS may also behave as a second messenger and modulate many downstream signaling pathways, including NF-κB and MAPK [19], [32]. In the present study, we sought to elucidate the role of ROS in PQ-treated neutrophils. We first measured apoptosis rates with flow cytometry and found that neutrophil survival was significantly prolonged following incubation with the higher concentration of PQ, and ROS accumulation was also increased. Notably, neutrophil ROS generation could be reversed by the specific NADPH inhibitor DPI or apocynin. This finding indicated that the effect of PQ on neutrophils was partly mediated through the promotion of ROS accumulation, which subsequently extended the neutrophil lifespan and resulted in persistent and severe tissue inflammation. However, these effects are concentration independent when the PQ concentrations were more than a certain level (≥5 μM).

We next investigated how ROS delayed neutrophil apoptosis by performing western blotting for p38 MAPK, Akt, and NF-κB. The results showed that both p38 MAPK and NF-κB pathways were involved in the regulation of PQ-induced neutrophil apoptosis delay, but Akt signaling was not. PQ might activate p38 MAPK and NF-κB through ROS accumulation. NF-κB translocation and transcriptional activity are dependent the degradation of IκB, which prevents NF-κB nuclear entry by covering the nuclear localization sequence [20], Therefore, we assessed the status of IκBα and found that IκBα degradation was dramatically induced by ROS accumulation following PQ treatment. Conversely, the NF-κB inhibitor PDTC significantly accelerated neutrophil apoptosis, a result that is consistent with previous studies showing that NF-κB can delay neutrophil apoptosis [33]–[35]. p38 MAPK also took part in the PQ-induced delay of neutrophil apoptosis, and this was attenuated by the p38 MAPK inhibitor SB203580. The prolonged neutrophil lifespan following PQ treatment was also reflected by increased expression of the antiapoptotic protein Mcl-1. p38 MAPK has a very short half life [36], [37], and the much more stable proapoptotic proteins of Mcl-1 tend to be dominant in the absence of survival signals. In our experiments, PQ-induced neutrophil survival was also decreased following treatment with Mcl-1 inhibitor SB200137. However, SB203580 could block both phosphorylation of p38 MAPK and Mcl-1, but SC200137 could only suppress p-Mcl-1 production, which demonstrated that the more stable Mcl-1 was mediated by p38 MAPK under PQ stimulus, and that these events occurred downstream of p38 MAPK. Nevertheless, Akt did not contribute to neutrophil survival following PQ treatment.

NF-κB and p38 MAPK are both robust inducers of proinflammatory cytokines that can facilitate cell death [32], [38]. Therefore, we further investigated the status of proinflammatory TNF-α, IL-1β, and IL-6, and found that PQ could induce production of all three, whereas the ROS inhibitor DPI suppressed their levels. This finding indicates that TNF-α, IL-1β, and IL-6 are involved in PQ's apoptotic effect toward neutrophils. A previous study revealed that cytokines can also promote ROS generation [39]. To verify whether this mechanism was also involved in PQ's effect on neutrophils, antibodies against TNF-α, IL-1β, and IL-6 were preincubated with the cultures to block their physiological actions. The results showed that ROS generation was suppressed by preincubation with TNF-α and IL-6 antibodies but not IL-1β. This finding indicates that TNF-α, IL-6, and ROS form a positive feedback circuit. On one hand, PQ-induced ROS accumulation could promote the production of TNF-α and IL-6 to trigger the tissue injury; on the other, increased TNF-α and IL-6 concentrations promote further ROS generation. This vicious cycle might subsequently result in an inflammatory cascade that aggravates tissue injury and could partially explain why the correlation between ROS generation and delayed neutrophil apoptosis was independent of PQ concentration.

In conclusion, our study elucidated the mechanism underlying PQs effect on neutrophil apoptosis. The results suggested that ROS have a central role in neutrophil survival. High PQ concentration induced ROS accumulation, which activated NF-κB and p38 MAPK signaling pathways, which promoted TNF-α and IL-6 production. In turn, TNF-α and IL-6 formed a positive feedback circuit and perpetuated further ROS generation. Clinically, this mechanism may explain why oxygen therapy exacerbates tissue injuries in PQ-poisoned patients, especially in the lung. Higher oxygen concentrations trigger the ROS–p38 MAPK/NF-κB–TNF-α/IL-6 positive feedback circuit and subsequently exacerbate symptoms. This finding may also stimulate research into novel treatments for PQ-poisoned patients.
